# *In Silico* Analysis of Single Nucleotide Polymorphism (SNPs) in Human *β*-Globin Gene

**DOI:** 10.1371/journal.pone.0025876

**Published:** 2011-10-20

**Authors:** Mohammed Alanazi, Zainularifeen Abduljaleel, Wajahatullah Khan, Arjumand S. Warsy, Mohamed Elrobh, Zahid Khan, Abdullah Al Amri, Mohammad D. Bazzi

**Affiliations:** Genome Research Chair Unit, Department of Biochemistry, College of Science, King Saud University, Riyadh, Saudi Arabia; University of South Florida College of Medicine, United States of America

## Abstract

Single amino acid substitutions in the globin chain are the most common forms of genetic variations that produce hemoglobinopathies- the most widespread inherited disorders worldwide. Several hemoglobinopathies result from homozygosity or compound heterozygosity to beta-globin (*HBB*) gene mutations, such as that producing sickle cell hemoglobin (HbS), *HbC*, *HbD* and *HbE*. Several of these mutations are deleterious and result in moderate to severe hemolytic anemia, with associated complications, requiring lifelong care and management. Even though many hemoglobinopathies result from single amino acid changes producing similar structural abnormalities, there are functional differences in the generated variants. Using *in silico* methods, we examined the genetic variations that can alter the expression and function of the *HBB* gene. Using a sequence homology-based Sorting Intolerant from Tolerant (SIFT) server we have searched for the SNPs, which showed that 200 (80%) non-synonymous polymorphism were found to be deleterious. The structure-based method via PolyPhen server indicated that 135 (40%) non-synonymous polymorphism may modify protein function and structure. The Pupa Suite software showed that the SNPs will have a phenotypic consequence on the structure and function of the altered protein. Structure analysis was performed on the key mutations that occur in the native protein coded by the *HBB* gene that causes hemoglobinopathies such as: *HbC* (E→K), *HbD* (E→Q), *HbE* (E→K) and *HbS* (E→V). Atomic Non-Local Environment Assessment (ANOLEA), Yet Another Scientific Artificial Reality Application (YASARA), CHARMM-GUI webserver for macromolecular dynamics and mechanics, and Normal Mode Analysis, Deformation and Refinement (NOMAD-Ref) of Gromacs server were used to perform molecular dynamics simulations and energy minimization calculations on *β*-Chain residue of the *HBB* gene before and after mutation. Furthermore, in the native and altered protein models, amino acid residues were determined and secondary structures were observed for solvent accessibility to confirm the protein stability. The functional study in this investigation may be a good model for additional future studies.

## Introduction

Hemoglobinopathies are the most frequently encountered inherited diseases resulting from mutations in and around the globin gene that produce either structural or biosynthetic defects. Several hundred mutations have been reported that produce structural variants of hemoglobin (Hb) or affect the rate of synthesis of one or more of the globin chains (thalassemia) resulting in an imbalance of the α/non-α ratio. Some of the variants exist at a polymorphic level in some populations while others are rare [1]. The World Health Organization (WHO) estimates for the years 2007–2009, that globally almost 5%, of the world's population are carriers (i.e. heterozygous) of a potentially pathological Hb mutation (2.9% for thalassaemia and 2.3% for sickle cell disease). Each year approx. 300,000 infants are born worldwide with sickle-cell anemia (SCA) (70%) or thalassemia syndromes (30%). Globally, the percentage of carriers of thalassemia is greater than that of carriers of HbS, but because of the higher frequency of the HbS gene in certain regions, the number of affected births is higher than that of thalassemics. Although over 700 structural hemoglobin variants have been identified, only three (Hb S, Hb C and Hb E) reach high frequencies. Some mutations result in a mild phenotype, while others produce severe clinical manifestations of the disease in individuals homozygous (SCA, *HbE* and *HbC* disease) or double heterozygous (*HbSC*, *HbS*/thalassaemia) for the mutations [2]. Studies have confirmed that carriers are healthy and asymptomatic and are protected against the lethal effects of malaria as they have an inborn resistance to the development of malaria. These hereditary anemias were originally confined to the tropics and subtropics, but due to the high rate of population movement, the spread is to almost all countries of the World, with high incidence rates in malaria endemic regions [3], [4], [5]. Although a single abnormal gene may protect against malaria, inheritance of two abnormal genes leading to the sickle cell disease (SCD) confers no such protection. Only a few Hb disorders have been well studied for the structural changes in the globin chains due to the mutation and the resultant functional abnormalities. Usually, a hemoglobinopathy resulting from a single amino acid substitution presents only one aberration in the primary and secondary structure. Mutation producing *HbE* also reduces the rate of synthesis, producing a condition referred to as *HbE*/*β*-thalassemia, which is common and presents an increasingly important health problem in many parts of Asia [6], [7]. The single nucleotide variations in the genome that occur at a frequency of more than 1% are referred to as single nucleotide polymorphisms (SNPs). In the human genome SNPs occur in just about every 3000 base pairs [8] and the frequency of occurrence of the different alleles differs in different populations.

The harmful SNPs for the *HBB* gene have not been predictable to date *in silico*. Therefore, to explore possible associations between genetic mutation and phenotypic variation, different algorithms like PupaSuite, SIFT and PolyPhen were used for prioritization of high-risk nonsynonymous single nucleotide polymorphisms (nsSNPs) in coding regions that are likely to have an effect on the function and structure of the protein. The frequent mutations producing hemoglobin variants i.e. *HbC* (E6K), *HbD* (E121Q), *HbE* (E26K) and *HbS* (E6V) were included in this study, in which 3D model structures of the mutant proteins were compared with the native protein structure. We further examined the native and mutant protein structures for solvent accessibility and secondary structure analyses. Our in-silico study further suggests the presence of additional deleterious mutations in *HBB* gene that may affect the structure and function of proteins with apparent roles in hemoglobinopathies and thalassemias.

## Materials and Methods

### Datasets

The clinical report of a patient (#0051421) from test directory of ARUP laboratory, USA was retrieved (http://www.aruplab.com/guides/ug/tests/0051421.jsp) where polymerase chain reaction (PCR) and florescence resonance energy transfer were used for disease identification. The test was performed pursuant to an agreement with Roche Molecular Systems, Inc. The blood samples (3 ml) were collected at 2–8°C. The four *ß*-globin gene missense mutations were detected i.e. *HbS*, *HbC*, *HbD* and *HbE* that have one amino acid change in the *ß*-globin chain. *HbS*, *HbD* and *HbC* cause an abnormal structure, while in the *HbE* mutation, there is a structural and biosynthetic abnormality since the mutation influences the splicing capability that results in reduced amount of *ß*-globin chain synthesis.

### Prediction of tolerated and deleterious SNPs using SIFT

**Figure 1:**
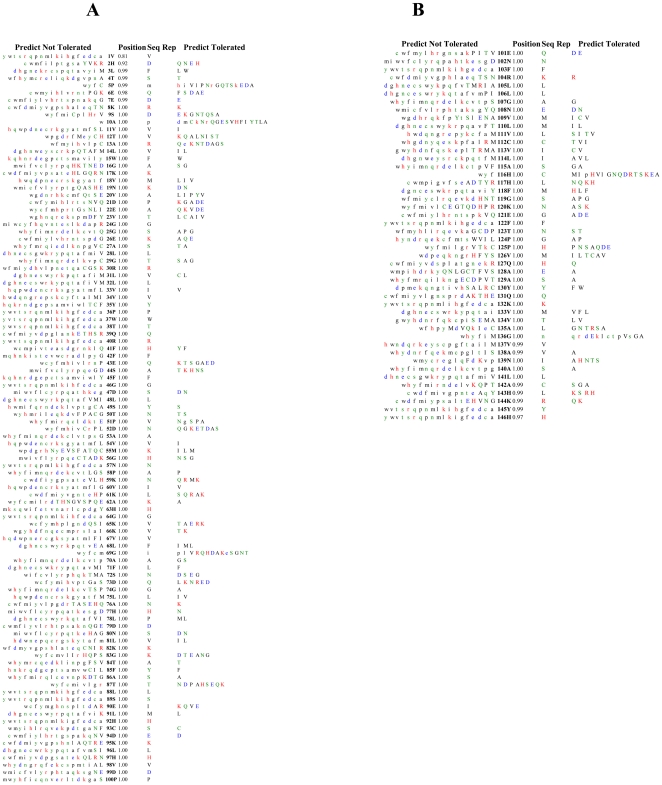
Sequence homology-based results from Sorting Intolerant from Tolerant (SIFT) server for SNPs. **a**) Predictions threshold for intolerance positions 1 through 100. **b**) Predictions threshold for intolerance positions 101 through 200. The type of amino acids in both figure are shown in color code i.e. Non polar (black), uncharged polar (green), basic (red), acidic (blue). Capital letters indicate amino acids appearing in the alignment, lower case letters result from prediction. ‘Seq Rep’ is the fraction of sequences that contain one of the basic amino acids. A low fraction indicates the position is either severely gapped or unalignable and has little information. Expect poor prediction at these positions.

Sorting Intolerant from Tolerant (SIFT, version 2) is a program that can identify if an amino acid substitution influences a protein function as well as a phenotypic change. It has been reported that SIFT can distinguish between functionally neutral and deleterious amino acid changes in mutagenesis studies and on human polymorphisms (http://blocks.fhcrc.org/sift/SIFT.html) [9]. SIFT analysis was based on algorithms to find homologous sequences using database SWISS-PORT version 51.3 and TrEMBL 34.3, by selecting median conservation sequence score 3.00. Threshold for intolerance is 0.05 or less shown in (Figure 1A–1B). Seq-Rep is the fraction of sequences that contain amino acids shown in color code: black (non polar); green (uncharged polar); red (basic); blue (acidic). A low fraction indicates the position is either severely gapped or unalienable and has little information.

### Prediction of functional modification of coding nsSNPs

PolyPhen (Phenotyping Polymorphism) software version 2.0.9 automatically predicts the consequence of an amino acid change on the structure and function of a protein on specific empirical rules on the sequence. PolyPhen input option are protein sequence, [10] attainment number P68871 or database ID/accession number combined with sequence position with amino acid variants AA_1_E and AA_2_K. The program calculates position-specific independent count (PSIC) scores for every variant and estimates the difference between the variant scores, the difference of >0.339 is detrimental. A study of closest contact with functional site showed HIS 2F with a distance 4.509 Å and charge change at exposed site substitution E→K with normal accessibility score 0.85 also mapped the substitution site to known protein 3D structures.

### Analyzing the molecular effect of SNPs

PupaSuite version 3.1 is an interactive web-server based genotyping SNPs analysis program for selection of relevant SNPs within genes, such as validation status frequency and population data putative functional properties of pathological SNPs disrupting potential transcription factor binding site and Exon/Intron boundaries. The SNPs effect [11] and PupaSuite version 3.1 in combination are utilized to locate annotations for both coding and non coding SNPs, in addition to annotations for the SWISS-PROT set of human disease mutations [12]. The input consists of a list of genes responsible for a particular pathway depending on specific biological function. The specific gene types recognize either Ensembl or other external database line GeneBank, European Molecular Biology Laboratory (EMBL) and SWISSPORT). SNP effect utilizes TANGO algorithm [13], [14] to search for cross-*ß* aggregation within peptide sequences as well as in denatured proteins. The FoldX is a quantitative stability evaluation software based on cellular and functional processing through sequence or structural analyses and generates single amino acid alterations to study the effect of coding nsSNPs on various phenotypic characteristics including protein structure and dynamics.

### nsSNPs location modeling on protein structure

The single amino acid polymorphism database (SAAP) [15] and dbSNPs were used to recognize the protein encoded by *HBB* gene (PDB ID: 4HHB) and identified single point mutation residue positions. The particular mutation residues concurred with the results of PolyPhen and SIFT server program. The mutant protein structure energy minimization was done through ANOLEA (Atomic Non-Local Environment Assessment), a server that performed energy calculations on a protein sequence, examined the “Non-Local Environment” (NLE) of every heavy atom in the molecule [16]. The energy of each Pairwise interaction in this non-local environment is obtained from a distance-dependent knowledge-based mean force potential resulting from a database that conducts energy calculations at the atomic level in protein structures. The calculations are performed on the non-local interactions among all the heavy atoms of the standard amino acids present in the molecule and provide an energy value for every amino acid of the protein as output energy profile. High-energy zones (HEZs) in the profile are associated with errors or with potential interacting zones of proteins. The server yields the structure in three dimensions, recognizing the high-energy amino acids in the protein. The energy calculations of mutation residues were done only on β2 chains of *HBB* gene (PDB ID: 4HHB). Overall, the total energy E/KT slightly differed before and after mutation (Table 1), however the total energy of *HBB* (4HHB) protein β2 chain was −612E/KT. The calculated energy offers details concerning the protein structure stability criteria, which indicate general understanding of differences at the structure level.

**Table 1:** Total energy of before and after mutation residue on *β*2 Chain B.

Name	Amino acids	PositionChromosome11	Before point mutation Energy E/KT units	After point mutation Energy E/KT units	Before Total protein EnergyE/KT units	After Total protein EnergyE/KT units
***HbC***	Glu→Lys (E→K)	6	−3.734	−3.033	−612	−608
***HbD***	Glu→Gln (E→Q)	121	−6.621	−1.980	−612	−607
***HbE***	Glu→Lys (E→K)	26	−2.100	1.611	−612	−575
***HbS***	Glu→Val (E→V)	6	−3.734	−2.825	−612	−607

Where “E” is the energy, “−E/KT” is the frequency that is proportional to exp, “T” is the temperature and “K” is the Boltzmann constant.

### *In silico* solvent accessibility of amino acid residue in protein structure

A web based server, Accessible surface area (ASA) was used that shows solvent accessibility of amino acid residues in proteins structures. The protein structure was retrieved from DSSP (database of secondary structure assignments) [15], [17], the program that calculates DSSP entries from PDB (protein data bank), the entry implementation of ASA view for all protein or individual chain. The criterion used was to add chain or PDB query in the input file, generating ASA View plot for co-ordinate or PDB file. The amino acid residues to the solvent demonstrated three types of solvent accessibility i.e., buried, partially buried and exposed indicating, low, moderate and high accessibility respectively [18], [19]. Secondary structure in particular is important for studying the relationship between amino acid and protein structure. The DSSP program describes secondary structure, geometrical appearances and solvent exposure of proteins in Protein Data Bank format.

### Molecular dynamics (MD) simulation & energy minimization

The molecular dynamics (MD) simulations were carried out by using bio molecular simulation program CHARMM, an academic research program widely used for macromolecular mechanics and dynamics with resourceful analysis and manipulation tools of atomic coordinates and dynamics trajectories. CHARMM-GUI (http://www.charmm-gui.org) [20], offers a web-based graphical user interface to analyze a range of input files and molecular systems to standardize and make use of common as well as advanced simulation techniques in CHARMM. Although in this simulation, the solvated system was neither minimized nor equilibrated, however 0.15 M ions can be added in the simulation box by specifying ions (KCl) and concentration (C). The numbers of ions are automatically established by the ion-accessible volume (*V*), the total charge of the system (*Q_sys_*), and by the positive ion (*z_+_*) valency, to neutralize the total system charge, (*z_+_N_+_−N_−_ = −Q_sys_*). The ion-accessible volume, *V*, is anticipated by subtracting molecular volume from the total system volume, selecting ion placing method of Monte Carlo. The initial configuration of ions was verified using short Monte Carlo (MC) simulations with a primitive model, for instance van der waals interactions. The solvation free energy is expressed as nonpolar and electrostatic contributions, but the nonpolar contribution is again partitioned into repulsive and dispersive contributions using the Weeks. To lessen the computational attempt, the free energy simulations are performed with few explicit solvent water molecules in close proximity to the solute, while the influence of the rest of the solvent mass is shown implicitly as an effective solvent boundary potential (SSBP). KCl was included to box to neutralize the overall negative charge of the structures. Molecular dynamics simulations were performed with a 2-fs time step at a constant temperature of 300 K and a constant pressure of 1 atm under periodic solvent boundary conditions. The Particle Mesh Ewald method [21] was utilized for electrostatics, and a 12 Å cutoff was applied for van der Waals interactions. The TIP3P water model [22] was used to model the solvent.

The mutations were studied using Pymol viewer, and energy minimization for 3D structures were carried out using NOMAD-Ref server [23]. This server utilizes Gromacs using forcefield for energy minimization according to the steepest descent, conjugate gradient and L-BFGS methods [24]. We also used the conjugate gradient method for 3D structure optimization and the deviation between the two structures was assessed by their RMSD values.

## Results

### Prediction of tolerated and deleterious SNPs using SIFT

The SIFT program evaluates the significance of an amino acid substitution influencing protein function design on sequence homology and the physical properties of amino acids and in combination with naturally occurring nonsynonymous polymorphisms altering phenotype by aligning paralogous and orthologous protein sequences. SIFT program was used to examine the tolerance and intolerance of a substitution among the SNPs. The predictions for positions from 1 to 100 and from 101 to 200 of amino acid residues threshold for intolerance index score of 0.05 are shown in (Figure 1A–1B) respectively. In both threshold intolerance has same ‘Seq-Rep’ is the fraction of sequences that contain one of the basic amino acids. A low fraction indicates the position is either severely gapped or unalienable, hence poor prediction is expected at this position. SIFT score were categorized as tolerant (0.201–1.00) or intolerant (0.051–0.10) and borderline (0.101–0.20) [9], [25]. About 150 out of 200 nsSNPs, indicated highly deleterious tolerance index score of 0.00, 3 indicated a score of 0.04 and other 3 showed a score of 0.05, 10 were reported to have a score of 0.02 while the rest of the 20 had a score of 0.01. These SNPs may perhaps have an impact on the protein function in the *HBB* gene. Out of total 200 nsSNPs in *HBB* gene only 20 nsSNPs suggested to be tolerant.

### Prediction of functional modification of coding nsSNPs

PolyPhen (Polymorphism and Phenotype) program was used to determine the protein structural modifications exhibiting potential impact of an amino acid substitution on the structure and function of the protein. SIFT results were also submitted as input to the PolyPhen server, PolyPhen server exploits both UniProtKB/UniRef100 non-redundant protein sequence and PDB/DSSP protein structure databases. The functional effect of amino acid changes can be shown by evaluating conservation where using PhyloPhen it was shown that accessing (Acc) number P68871, mutant position is 6, amino acid substitution of (AA1)E→K (AA2). This prediction variant was found to be benign based on the alignment. The PSIC score calculation difference was 0.0339, near the functional site of HIS 2F with distance 4.509 Å. The charge alteration at the exposed site of substitution E→K, with normed accessibility is 0.085, based on physicochemical properties and proximity of the substitution to predict functional domains or structures. The PSIC scores between the two amino acid variants ranged from +0.482 to +0.82. The selected best score between one and two was 0.339. The multiple alignments around substitution position flanks was 25, mapping substitution site to known protein 3D protein structures from the PQS database, initial number of structure was 500 and final number of the structure was 480. PolyPhen scores were allocated probably damaging (2.00 or more), possibly damaging (1.40–1.90), potentially damaging (1.20–1.50), benign (0.00–0.90) and borderline (1.00–1.20). From a total of 200 protein sequences of nsSNPs submitted to the SIFT as well as submitted to the PolyPhen server, out of 200 nsSNPs, 120 nsSNPs (55%) suggested to be damaging with a PSIC score greater than 2.1 and may have an effect on the protein quaternary structure and their functionality. The remaining 100 nsSNPs were found to be non-damaging by PolyPhen. It is noted that 125 nsSNPs observed to be deleterious by SIFT program were also predicted to be damaging by PolyPhen server.

### Analyzing the molecular effect of SNPs

The PupaSuite is a World Wide Web tool for selecting SNPs with potential phenotypic effects; this server aims to offer a platform for predicting the consequence of coding nsSNPs on the protein structure and function. The SNPs effect and PupaSuite databases are used together to locate annotations for both coding and non-coding SNPs, as well annotations for the SWISSPORT for numerous human disease mutations. We submitted *HBB* gene in PupaSuite and selected a particular type of gene identifiers using Ensembl. Out of 200 SNPs, 100 nsSNPs were shown to affect the exonic splicing enhancers. 1 SNP in mRNA disrupted the exonic splicing enhancers, 2SNPs (synonymous) were predicted to disrupt the exon splicing enhancers, and 36 nsSNPs were pathological SNPs and were implicated in the cellular processing. About 17 nsSNPs disrupted the exonic splicing silencers, 1 SNP in the intronic section was implicated in intronic and exon junctions and 9 nsSNPs were implicated in dynamic and protein structure. The selective acting codon level SNPs was found by selecting pathological mutation predicted by selective constraints (v = dN/dS) then choose omega (v). The ratio of ns synonymous (v = dN/dS) is a significant pressure at the protein level. Along neutral mutations (v = 1), there is two types of selection mode 1st, purifying selection (V<1), 2nd diversifying positive selection. In number of 2 SNPs with ID rs33973589 and rs33935383 showed a selective pressure (v_slr: 0.0000) (P>0.05); v_bay: 0.121 (M8) at a codon level were obtained throughout Phylogenetic Analysis by Maximum Likelihood (PAML) programme [26]. The SNPs were considered to be deleterious resulting in both functional and structural alteration of the protein by SIFT, PupaSuite and PolyPhen server.

### nsSNPs location modeling on protein structure

**Figure 2:**
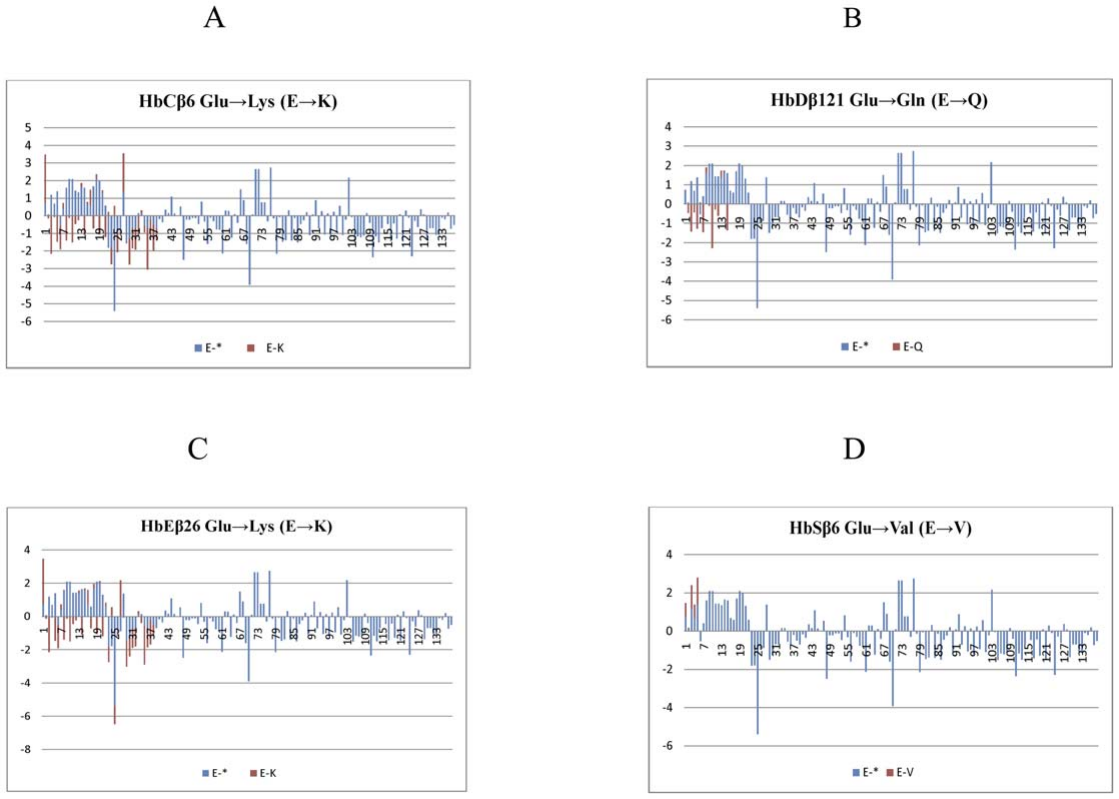
Human *ß*-globin protein structure stability alterations following mutation. **a**) *HbCβ*6 E Glu to mutated protein K Lys, **b**) *HbDβ*121 E Glu to mutated protein Q Gln Lys, (**c**) *HbEβ*26 E Glu to mutated protein K Lys, (**d**) *HbSβ*6 E Glu to mutated protein V Val.

For the routine prediction of protein stability alterations upon single-site mutations a neural-network-based web server I-Mutant version 2.0 was exploited [27]. For this prediction protein 3D structure is required [28]. The tool was applied on a data set extracted from ProTherm (Ex. Collection of numerical data of thermodynamic parameters such as Gibbs free energy change, enthalpy change, heat capacity) [29] efficiently indicates whether a protein mutation affects the stability of the protein structure or not. This server also provides the scores of free energy change predictions calculated with the energy-based FOLD-X tool. By incorporating the FOLD-X approximation with those of I-Mutant, an precision of 93% on one third of the database can be accomplished, thus making I-Mutant a helpful tool for protein design and mutation. The following mutations in *HBB* gene resulted in *HbC* [*ß*6, Glu→Lys, E→K)], *HbD* [(*ß*121, Glu→Gln (E→Q)], *HbE* [(*ß*26, Glu→Lys (E→K)] and *HbS* [(*ß*6, Glu→Val (E→V)]. Amino acid in wild type protein (WT) should be E (Glu), however new Amino acids are substituted in its place after mutations such as E→K, E→Q, E→K, E→V. The prediction effects of new amino acid caused Support Vector Machine (SVM2) and DGG stability change upon point mutation and the reliability index differed (Table 2).The stability changes after mutation from native protein to mutated protein are shown in (Figure 2A–2D).

**Table 2:** Protein Stability Changes upon Mutations.

Name	Position	WT	NEW	pH	T	SVM2Prediction Effect	RI	DDG ValuePrediction Kcal/mol
***HbC*** Glu→Lys (E→K)	6	E	K	7.0	25	Decrease	8	−0.50
***HbD*** Glu→Gln (E→Q)	121	E	Q	7.0	25	Decrease	7	−0.45
***HbE*** Glu→Lys (E→K)	26	E	K	7.0	25	Decrease	9	−1.10
***HbS*** Glu→Val (E→V)	6	E	V	7.0	25	Increase	1	0.35

Where, “WT” is the wild-type amino acid in the protein, “NEW” is the new amino acid after Mutation, “SVM2/DGG” is the stability (decrease/ increase), “RI” is the Reliability Index, and “T” is the temperature in celsius degrees.

### *In silico* solvent accessibility of amino acid residue in protein structure and MD simulation of native protein and mutants residues

**Figure 3:**
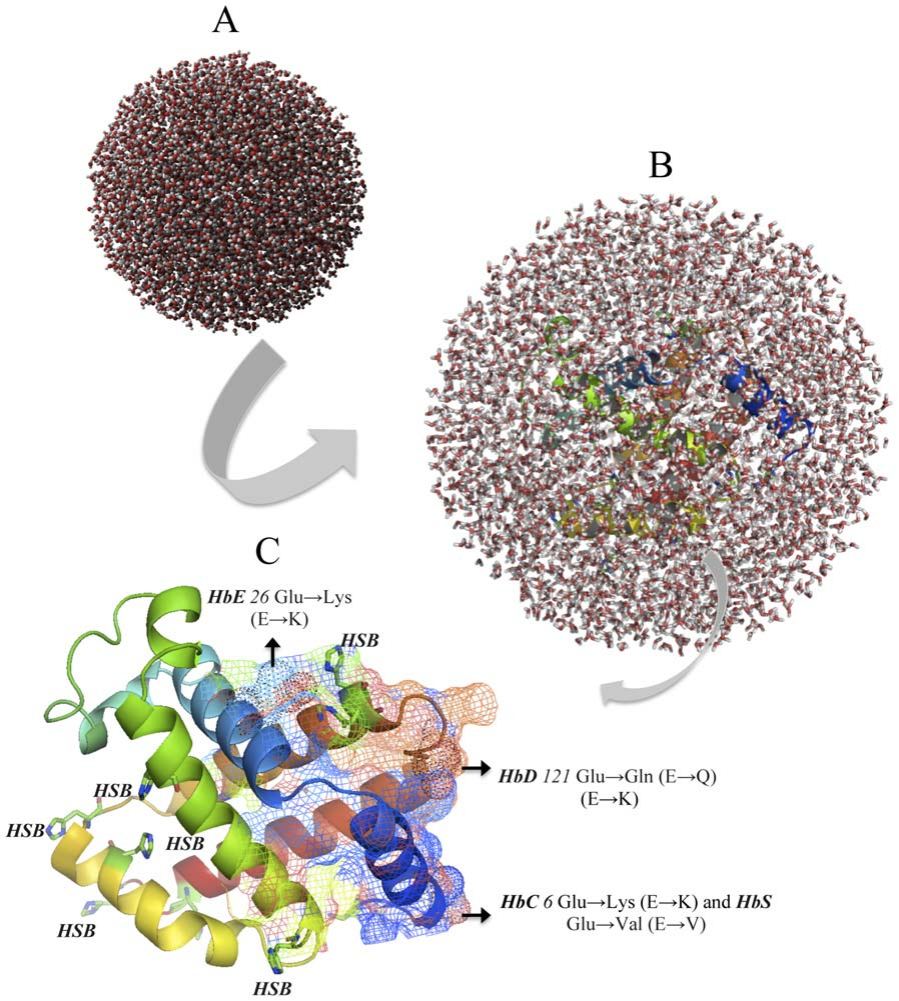
The ß-chain of the HHB protein's molecular dynamics (MD) simulation showing truncated octahedron boundary explicit water solvated and hydrogen atoms in color. The MD simulations system used in calculations are; **a**) water box without the protein, **b**) water box surrounding the entire protein (middle) and **c**) tertiary structure of the wild type *ß*-chain of HHB protein showing disease causing mutations i.e. green *HbE*26 Glu→Lys (E→K), orange *HbD*121 Glu→Gln (E→Q), blue *HbC*6 Glu→Lys (E→K) and *HbS*6 Glu→Val (E→V) on 3 helixes using predict mesh modeling. The visual inspection also allow to identify the side chain of a histidine residue involved in the hydrogen bonding with surrounding molecules and in that case the δ nitrogen of the histidine (HSB) is protonated residue.

**Figure 4:**
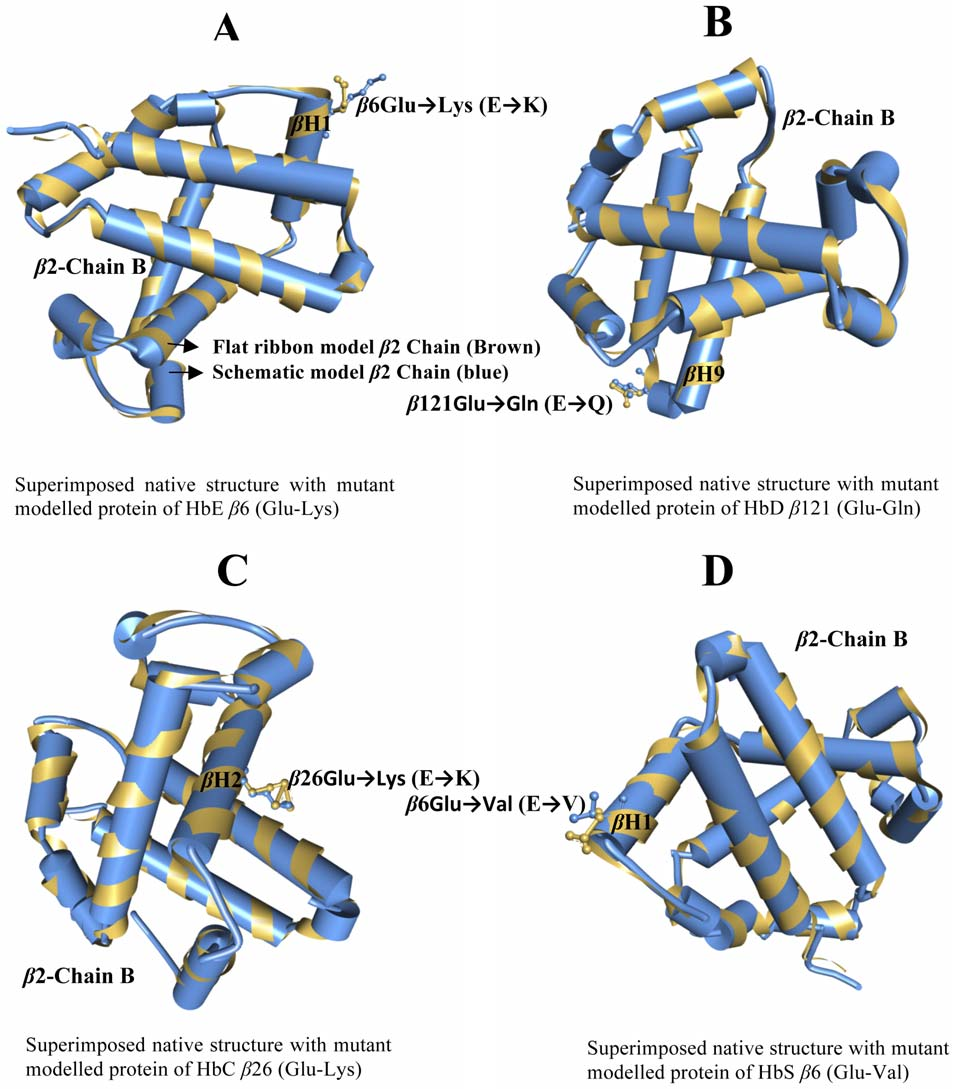
The superimpose alignments of crystal structure of human deoxyhaemoglobin native protein structure (4HBB) with mutant modelled protein i.e. *HbE*, *HbD*, *HbC* and *HbS*. The structural modeling of proteins are flat ribbon model1 *β*2 Chain B (Brown) and Schematic model2 *β*2 Chain (blue). Four different amino acid mutations occur in the *β*2 Chain B, **a**) *HbE* contains *β*6Glu→Lys (E→K) located in the *β* Helix1, **b**) *HbD* contains *β*121Glu→Gln (E→Q) on located in the *β* Helix9, **c**) *HbC* contains *β*26Glu→Lys (E→K) in the *β* Helix2 and **d**) *HbS* contains *β*6Glu→Val (E→V) in the β Helix1.

Point mutations causing amino acid alteration can drastically modify the stability of a protein structure; hence the modeling of protein structural information is necessary for absolute understanding of its functionality. The deleterious nsSNPs were obtained from database of single nucleotide polymorphism (dbSNP) (http://www.ncbi.nlm.nih.gov/SNP) [30], and from the single amino acid polymorphism database (SAAPdb) (http://www.bioinf.org.uk/saap/db/) [31], which is collected data on SNPs from both dbSNP and (Human Genome Variation database) HGVBASE that displays the data onto the translated regions of the gene to establish whether the mutation is in a region of the gene translated to protein (in exon) and, if so, whether it is resulting in a missense mutation in the protein. The available protein structure of HBB gene showed PDB ID: 4HHB and *HBB* mutations showing β-Hemoglobinopathies on the ß-chain were following (1) *HbC β*6; AA Glu→Lys (E→K), (2) *HbD β*121; AA Glu→Gln (E→Q), (3) *HbE β*26; Glu→Lys (E→K), and (4) *HbS β*6; Glu→Val (E→V). The mutations for 4HHB at the corresponding positions were achieved using SWISS-PORT viewed separately to obtain altered model structures. The energy minimizations were achieved by YASARA and NOMAD-Ref Gromacs server using force field energy for the native type protein 4HHB and mutant type protein structures from the protein model RMSD. The total energy of native structure 4HHB and mutant model structure residues were calculated (Table 3) and showed range from 0.25 to 0.50 RMSD native and mutant protein structure, respectively. The total energy for the native type protein structure (4HHB) following energy minimization was −334380.5 kJ/mol (score; 0.69), where as prior to energy minimization it was 29644578.1 kJ/mol (score: −3.31). Then using the CHARMM-GUI server that offers graphical user interface (GUI) for MD simulations, the consequence of mutations were studied using classical molecular dynamics approach for analyzing both the native and the fetal mutations through long simulations in explicit solvent as well as investigating the differences in dynamics and stability which were investigated in the ß chain of 4HBB protein and to compute the free energy. The solvator was used to create a realistic aqueous solvent environment around the ß chain of 4HBB protein with water. The solvator determines the dimension of system with octahedral shapes of water box fitting to fully solvate the molecule with edge distance 10.0 as shown in (Figures 3A–3C). The energy minimizations studies were done by NOMAD-Ref server for the native type protein (4HHB) and the mutant type structures, the total energy for the native type protein structure (4HHB) following energy minimization was −334380.5 kJ/mol (score; 0.69), where as prior to energy minimization it was 29644578.1 kJ/mol (score: −3.31). The superimposed structures of the native (4HHB) with mutant type proteins are shown in Figure 4A–4D.

**Table 3:** RMSD and total energy of native structure (4HHB) and mutant modeled structures.

Name	Aminoacid variant	RMSD native and mutant protein structure	Total energy after minimization KJ/mol
***HbC***	Glu→Lys (E→K)	0.50	−334383.20
***HbD***	Glu→Gln (E→Q)	0.42	−337822.06
***HbE***	Glu→Lys (E→K)	0.35	−336831.04
***HbS***	Glu→Val (E→V)	0.25	−333364.02

The total energy of native structure (4HHB) after energy minimization is −334380.5 kJ/mol (score: 0.69) and before energy minimization it was 29644578.1 kJ/mol (score: −3.31).

**Figure 5:**
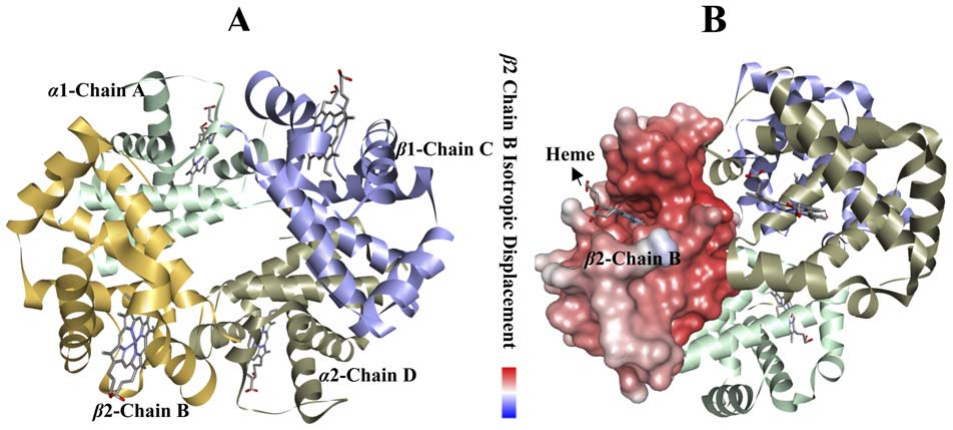
The crystal structure of human deoxyhaemoglobin at 1.74 A resolution. The haems are located toward the proximal region; the partition between the mean planes of N (porphyrin) and C (porphyrin) being 0.16(6) A and 0.10(6) A, respectively at the alpha and beta haems. At the alpha haems, the normal's to the average pyrrole planes are skewed evenly toward the haem centre, by about three degrees relative to the haem normal, and there is a folding of about four degrees of the haem about an axis running between the methene carbons that are between the pyrrole rings bearing like-type side-chains. At the beta haems, there is no such folding (Figure 5a) having α1-Chain A (pale green), *β*2-Chain B (yellow), *β*1-Chain C (violet) and α2-Chain D (grey) which has four haems in each chain but this study was focused on *β*2-Chain B similar to the isotropic displacement model (Figure 5b).

The crystal structure of human deoxyhaemoglogin at 1.74 Å resolutions (Figure 5A), showed four chains, i.e. α chain a, β chain b, α chain c and β chain d, where as each chain is shown in different colors. Another protein model structure of β2 chain using modeling of isotropic displacement with Heme binding (Figure 5B), whereas the superimposed structures of the native and mutant amino acid of *HbC*, *HbD*, *HbE* and *HbS* at their corresponding positions are shown in Figure 4A–4D. The superimposed native structure with mutant model proteins of *HbE β*6 (Glu→Lys) positioned on the *β*-Helix1, *HbD β*121 (Glu→Gln) located at the *β*-Helix9, *HbC β*26 (Glu→Lys) placed on *β*-Helix2 and *HbS β*6 (Glu→Val) located at *β*-Helix1. The prediction of residue solvent accessibility can assist in better understanding the relationship between structure and sequence. 3D analyses of a mutated protein structure were used by the project HOPE (http://www.cmbi.ru.nl/hope) who's mission is to build an automatic mutant analysis server that can provide insight in the structural effects of a mutation [32]. Therefore, the first mutation of *HbE* (Glu→Lys), each amino acid has its own specific size, charge, and Hydrophobicity-value. The original wild type residue and newly established mutant residue sometimes vary with regard to this property. The mutant residue is molecularly larger than the wild type residue. The wild type residue was negatively charged, where in case of mutant this residue was positively charged. The mutant amino acid properties are difference in charge between the wild type and mutant amino acid. The mutation introduces the opposite charge at this position. This possibly disrupts contacts with other molecules. The wild type and new mutant amino acid differ in molecular size. The residue is located on the surface of the protein; mutation of this residue can disturb interactions with other molecules or other parts of the protein. The second mutation of *HbD* (Glu→Gln), the mutant residue is smaller than the wild type residue. The wild type residue was negatively charged and the mutation residue is neutral. The report will evaluate the effect of the mutation, contacts made by the mutated residue, structural domain in which residue is located, modifications on this residue and known variations for these residues. On structure the mutation introduces Glutamine at the position. Glutamines are very flexible and can disturb the required rigidity of the protein on this position. The amino acid properties are a difference in charge between the wild type and mutant amino acid. The charge of the wild type residue will be lost; this can cause loss of interactions with other molecular residues. The wild type and new mutant amino acid differ in size, the mutant residue is smaller, and this might lead to loss of interactions. The Hydrophobicity of the wild type and new mutant residue differs. This mutation introduces a more hydrophobic residue at this position. This can in result in loss of hydrogen bonds and disturbs correct folding. In the third mutation of *HbC* (Glu→Lys), the mutant residue is larger than the wild type residue. The wild type residue and newly introduced mutant residue often differ in these properties. The wild type residue was negatively charged, the mutant residue is positively charged. The report will evaluate the effect of the mutation contacts made by the mutated residue, structural domains in which the residue is located, modification on this residue and known variants for this residue. The contact size residue difference between wild type and new mutant residue makes that the new residues is not in the correct position to make the same hydrogen bond as the original wild type residue did. The difference in charge will disturb the ionic interaction made by the original wild type residue. The wild type residue is much conserved, but a few other residues types have observed at this position too. Our mutant residue was among the residues at this position observed in other sequences. The difference in charge will disturb the ionic interaction made by the original wild type residue. This mutation introduces the opposite charge at this position. This possibly disrupts contacts with other molecules. The wild type and new mutant amino acid differ in size. The mutant residue is bigger than the wild type on the surface of the protein, mutation of this residue can disturb interaction with other parts of the protein. And in the fourth mutation of *HbE* (Glu→Val), this mutant residue is smaller than the wild type residue. The wild type residue was negatively charged, the mutant residue is neutral. The mutant residue was more hydrophobic than the wild type residue. The wild type residue is not conserved at this position. Our mutant residue was not among types observed at this position in other homologous sequences, which indicate that the mutation is possibly damaging to the protein. Amino acid properties are a difference in charge between the wild type and mutant amino acid. The charge of the wild type residue is lost by this mutation. This can cause loss of interactions with other molecules. The wild type and new mutant amino acid differ in size and was smaller than the wild type. This will cause a possible loss of external interactions.

## Discussion

Single nucleotide polymorphisms (SNPs) are the common form of genetic variations among individuals, and are thought to be accountable for the majority of inherited traits, including a large portion of inherited disease vulnerability. The link between a single nucleotide change and monogenic disease has been reported for a number of cases, and about 1000 proteins are identified to be associated with this process. Many human SNPs that are now recognized (in excess of 4-million unique SNPs) (http://www.ncbi.nlm.nih.gov/SNP/index.html), along with the genome sequence and other proteome information, provide an opportunity for a much broader understanding of the association between genotype and phenotype at this level. Nonetheless, to date the complete mechanisms by which a SNP may result in a phenotypic change are for the most part are unknown. About 2% of the all known single nucleotide variants associated with monogenic disease are non-synonymous SNPs in protein-coding regions (i.e., SNPs that alter a single amino acid in a protein molecule). As a result, it is anticipated that this class of SNPs are related to complex inherited disease traits, even though 900 hemoglobin variants have been mentioned [33], merely ∼25% of those have been fully documented either as being without clinical manifestation or accountable for considerable disorders. The sickle cell diseases (SCD) are inconsistent in their appearance due to differences in the genetic composition and the environmental exposure of the affected individual. While the precise genes that influence SCD clinical presentations are yet to be identified, broad understanding of the pathophysiology of SCD indicates that genes implicated in several mechanisms might have epistatic potential in SCD. The phenotypic affect will present a disease risk, despite the fact that thousands of SNPs are likely to exist in the human population from only small subset of variation. Since nsSNPs, among the SNPs variations likely cause a mutation of the encoded amino acids are thought to have the greatest influence on protein function. An important feature of post-genome biology is predicting phenotypes arising due to nsSNPs. Progress in the study of genetic variation perhaps will show the way to a better understanding of the resultant phenotypic variations amongst individuals with an endeavor towards drug design and development [34]. Previously, the HBB gene (PDB ID: 1DXT) have been analyzed for native and mutant protein structures [35]. However, in the present study we used a different protein (PDB ID: 4HHB) of HBB gene based on the clinical report of a patient (#0051421) from the test directory of ARUP laboratory, USA. A computational approach was exploited to study systematic analysis of SNPs by means of PupaSuite, PolyPhen and SIFT. Therefore, an effort was made to identify SNPs that can modify the function and expression of the *HBB* gene causing hemoglobinopathies and recommend computational strategy for the systematic analysis of SNPs. PolyPhen scores were assigned probably damaging (2.00), potentially damaging (1.20–1.50), perhaps damaging (1.40–1.90), benign (0.00–0.90) and borderline (1.00–1.20) [36]. There were 200 protein sequences of nsSNPs submitted to the SIFT as well as to the PolyPhen server, out of these 200 nsSNPs, 120 nsSNPs (55%) were deleterious. These nsSNPs with a PSIC score of more than 2.1 probably alter the protein structure and functionality. SIFT is a program designed on the levels of conservation among the species. Information regarding the common position of the amino acid substitution relative to critical structural and functional characteristics provide further understanding data to the evolutionary conservation. The predictions for positions 1 to 100 and 101 to 200 of residues threshold for intolerance index score of 0.05 are shown in Figure 1A–1B. In both threshold intolerances have same ‘Seq Rep’ a fraction of sequences that contain one of the basic amino acid. ‘Seq Rep’ column correspond to the percentage of sequences in the alignment, which have an amino acid (not an insertion/deletion) at the corresponding position. A low fraction indicates that the position is either severely gapped or unalienable and we also expect poor prediction at this position which is based on already established classification [25]. SIFT scores were classified as tolerance (0.201–1.00), intolerant (0.051–0.10), or borderline (0.101–0.20) Among the 200 nsSNPs, 150 had highly deleterious tolerance index score of 0.00A variation within functional domain in both oxygen binding and protein interactive region is likely to have an impact than the one in a non-critical location. Based on a decision tree, PolyPhen exploits different species information using structural parameters. The assessment of non-neutral SNPs is largely dependent on phylogenetic informatics by means of correlation with residue conservation to a certain degree with structural methods (PolyPhen). By utilizing SIFT and PolyPhen (PupaSuite) we analysed the functional significance of nsSNPs that were shown to be deleterious and damaging. ESSs (Exonic splicing silencer) are sequence elements that participate in alternative splicing and also play a part in splice site selection [37]. Nonsense and missense mutations can modify exonic splicing enhancers (ESEs) resulting in the splicing machinery to fail to spot the mutant exon, consequently causing drastic changes in the structure of the gene product [38]. In silico procedures are endowed with a valuable tool with initial step of locating any mutation supposed to result in aberrant RNA processing. ESEs are rich in alternative and constitutive exons, they act as binding sites for Ser/Arg-rich proteins (SR proteins), and a group of conserved splicing factors taking part in various steps of the splicing pathway [39]. Mutation study for the *HBB* gene was conducted for four ß-haemoglobinopathies, *HbC* [*β*6; AA Glu→Lys (E→K)], *HbD* [*β*121; AA Glu→Gln (E→Q)], *HbE* [*β*26; Glu→Lys (E→K)], and *HbS* [*β*6; Glu→Val (E→V)]. In this study, by using solvent accessibility and secondary structure analysis we further assessed the structural significance of native and mutant protein models of the *HBB* gene.

The classical molecular dynamics approach was also applied for studying native and fetal mutations using simulations in explicit solvent and examining the differences in dynamics and stability of the ß chain of HBB protein. The energy minimizations studies of the native type protein (4HHB) and the mutant type structures showed that the total energy of the native type protein structure after energy minimization was −334380.5 kJ/mol (score; 0.69), which was 29644578.1 kJ/mol (score: −3.31) prior to energy minimization. The solvator was also implemented to create a realistic aqueous solvent environment around the ß chain of the HBB protein to determine the dimension of system with octahedral shapes of water box (Figure 2A–2B). The tertiary structure of the wild type ß-chain of HHB protein showed disease causing mutations i.e. green *HbE*26 Glu→Lys (E→K), orange *HbD*121 Glu→Gln (E→Q), blue *HbC*6 Glu→Lys (E→K) and *HbS*6 Glu→Val (E→V) on 3 helixes using predict mesh modeling. Histidine residue in the model is involved in the hydrogen bonding with surrounding molecules and the δ nitrogen of the histidine (HSB) in the structure is also protonated residue (Figure 2C). Considered as a discriminating feature in disease-associated nsSNPs, solvent accessibility was found to be present at buried sites [40]. By means of evolution-based strategy using SIFTS, structure-based strategy using PolyPhen and deducing the molecular phenotype effects of SNPs using PupaSuite will be helpful in choosing SNPs that are expected to have likely functional implication, eventually resulting in an individual's vulnerability to haemoglobinopathy by *HBB* gene. Our results from this investigating indicate the procedure of computational algorithms offer an alternative strategy to choose SNPs targets by considering role of SNPs on the functional attributes or molecular phenotype of a protein. *In silico* approaches based on these investigations will surely assist in our understanding of the inheritance of complex human diseases.

In conclusion, our results showed that the analysis of four different SNPs on the protein structure by *in silico*, *HbC* [*ß*6, Glu→Lys, E→K)], *HbD* [(*ß*121, Glu→Gln (E→Q)], *HbE* [(*ß*26, Glu→Lys (E→K)] and *HbS* [(*ß*6, Glu→Val (E→V)], the 1^st^ mutation *HbE* (Glu→Lys) of this residue can disturb interactions with other molecules or other parts of the protein. Whereas the 2^nd^ mutation *HbD* (Glu→Gln) residue have more hydrophobic residue at this position that result in loss of hydrogen bonds and disturb correct folding. Similarly, 3^rd^ mutations *HbC* (Glu→Lys) showed a difference in charge that will disturb the ionic interaction of the original wild type residue, which could possibly disrupts contacts with other molecules as well as disturb the interaction with the other parts of the protein. The 4^th^ mutation *HbE* (Glu→Val) was not among the types usually observed at this position in other homologous sequences; therefore this mutation is possibly damaging to the protein and will cause possible loss of external interactions. The study also analyzed *in silico* solvent accessibility of amino acid residues in protein structure, nsSNPs location modeling on protein structure, analyzing the molecular effect of SNPs, prediction of functional modification of coding nsSNPs, prediction of tolerated and deleterious SNPs using SIFT and based on such methods indicated that they can prove to be very useful for novel drug targets for the relevant protein structure and also these models built in this work would be applicable for predicting the deleterious nsSNPs which would be helpful for further future genotype–phenotype research as well as currently ongoing pharmacogenetics studies. The functional analysis in this study may perhaps exhibit a good model for further research activities in genetically inherited disease.

## References

[1] Weatherall DJ, Clegg JB (2001). The Thalassaemia Syndromes, 4th ed.

[2] Driscoll MC (2007). Sickle Cell Disease.. Pediatr in Rev.

[3] Piel FP, Patil AP, Howes RE, Nyangiri OA, Gething PW (2010). Global distribution of the sickle cell gene and geographical confirmation of the malaria hypothesis.. Nat Commun.

[4] Livingstone FB (1985).

[5] Barlow-Stewart K, Emery J (2007). Genetics in Family Medicine: The Australian Handbook for General Practitioners..

[6] Fucharoen S, Winichagoon P (1997). Hemoglobinopathies in Southeast Asia: molecular biology and clinical medicine.. Hemoglobin.

[7] Rees DC, Styles L, Vichinsky EP, Clegg JB, Weatherall DJ (1998). The hemoglobin E syndromes.. Ann N Y Acad Sci.

[8] Cargill M, Altshuler D, Ireland J, Sklar P, Ardlie K (1999). Characterization of single nucleotide polymorphisms in coding regions of human genes.. Nat Genet.

[9] Ng PC, Henikoff S (2001). Predicting deleterious amino acid substitutions.. Genome Res.

[10] Ramensky V, Bork P, Sunyaev S (2002). Human non-synonymous SNPs: server and survey.. Nucleic Acids Res.

[11] Reumers J, Maurer-Stroh S, Schymkowitz J, Rousseau F (2006). SNP effect v2.0: a new step in investigating the molecular phenotypic effects of human non-synonymous SNPs.. Bioinformatics.

[12] Conde L, Vaquerizas JM, Dopazo H, Arbiza L, Reumers J (2006). PupaSuite: finding functional single nucleotide polymorphisms for large-scale genotyping purposes.. Nucleic Acids Res.

[13] Fernandez-Escamilla AM, Rousseau F, Schymkowitz J, Serrano L (2004). Prediction of sequence dependent and mutational effects on the aggregation of peptides and proteins.. Nat Biotechnol.

[14] Rousseau F, Schymkowitz J, Serrano L (2006). Protein aggregation and amyloidosis: confusion of the kinds?. Curr Opin Struct Biol.

[15] Cavallo A, Martin AC (2005). Mapping SNPs to protein sequence and structure data.. Bioinformatics.

[16] Melo F, Feytmans E (1998). Assessing protein structures with a non-local atomic interaction energy.. J Mol Biol.

[17] Kabsch W, Sander C (1983). The DSSP program.. Biopolymers.

[18] Gilis D, Rooman M (1996). Stability changes upon mutation of solvent accessible residues in proteins evaluated by database derived potentials.. J Mol Biol.

[19] Gilis D, Rooman M (1997). Predicting protein stability changes upon mutation using database-derived potentials: solvent accessibility determines the importance of local versus non-local interactions along the sequence.. J Mol Biol.

[20] Jo S, Kim T, Iyer VG, Im W (2008). CHARMM-GUI: A Web-based Graphical User Interface for CHARMM.. J Comput Chem.

[21] Hockney RW, Eastwood JW (1981). Computer Simulation Using Particles.

[22] Jorgensen WL, Chandrasekhar J, Madura JD, Impey RW, Klein ML (1983). Comparison of simple potential functions for simulating liquid water.. J Chem Phys.

[23] Lindahl E, Azuara C, Koehl P (2006). NOMAD-Ref: visualization, deformation and refinement of macromolecular structures based on all-atom normal mode analysis.. Nucleic Acids Res.

[24] Delarue M, Dumas P (2004). On the use of low-frequency normal modes to enforce collective movements in refining macromolecular structural models.. Proc Natl Acad Sci.

[25] Xi T, Jones IM, Mohrenweiser HW (2004). Many amino acid substitution variants identified in DNA repair genes during human population screenings are predicted to impact protein function.. Genomics.

[26] Massingham T, Goldman N (2005). Detecting amino acid sites under positive selection and purifying selection.. Genetics.

[27] Capriotti E, Fariselli P, Casadio R (2005). I-Mutant2.0: predicting stability changes upon mutation from the protein sequence or structure.. Nucleic Acids Res.

[28] Berman HM, Westbrook J, Feng Z, Gilliland G, Bhat TN (2000). The Protein Data Bank.. Nucleic Acids Res.

[29] Gromiha MM, An J, Kono H, Oobatake M, Uedaira H (2000). ProTherm, version 2.0: thermodynamic database for proteins and mutants.. Nucleic Acids Res.

[30] Stephen TS, Minghong W, Karl S (1999). dbSNP—Database for Single Nucleotide Polymorphisms and Other Classes of Minor Genetic Variation.. Genome Res.

[31] Hurst JM, McMillan LE, Porter CT, Allen J (2009). The SAAPdb web resource: a large-scale structural analysis of mutant proteins.. Hum Mutat.

[32] Venselaar H, Te-Beek TA, Kuipers RK, Hekkelman ML (2010). Protein structure analysis of mutations causing inheritable diseases. An e-Science approach with life scientist friendly interfaces.. BMC Bioinformatics.

[33] Hardison RC, Chui DH, Giardine B, Riemer C, Patrinos GP (2002). HbVar: a relational database of human hemoglobin variants and thalassemia mutations at the globin gene server.. Hum Mutat.

[34] Wang Z, Moult J (2001). SNPs, protein structure and disease.. Hum Mutat.

[35] Doss CGP, Rao S (2009). Impact of single nucleotide polymorphisms in HBB gene causing haemoglobinopathies: in silico analysis.. New Biotechnology.

[36] Savas S, Kim DY, Ahmad MF, Shariff M, Ozcelik H (2004). Identifying functional genetic variants in DNA repair pathway using protein conservation analysis.. Cancer Epidemiol Biomarkers Prev.

[37] Fairbrother WG, Chasin LA (2000). Human genomic sequences that inhibit splicing.. Mol Cell Biol.

[38] Cartegni L, Chew SL, Krainer AR (2002). Listening to silence and understanding nonsense: exonic mutations that affect splicing.. Nat Rev Genet.

[39] Graveley BR (2000). Sorting out the complexity of SR protein functions.. RNA.

[40] Ferrer-Costa C, Orozco M, de la Cruz X (2002). Characterization of disease-associated single acid polymorphisms in terms of sequence and structure properties.. J Mol Biol.

